# Metabolic Gene Expression in the Muscle and Blood Parameters of Broiler Chickens Stimulated In Ovo with Synbiotics

**DOI:** 10.3390/ani10040687

**Published:** 2020-04-15

**Authors:** Aleksandra Dunislawska, Maria Siwek, Anna Slawinska, Adam Lepczynski, Agnieszka Herosimczyk, Pawel A. Kolodziejski, Marek Bednarczyk

**Affiliations:** 1 UTP, Department of Animal Biotechnology and Genetics, University of Science and Technology, 85-084 Bydgoszcz, Poland; siwek@utp.edu.pl (M.S.); slawinska@utp.edu.pl (A.S.); marbed13@op.pl (M.B.); 2 Department of Physiology, Cytobiology and Proteomics, West Pomeranian University of Technology, 71-270 Szczecin, Poland; adam.lepczynski@zut.edu.pl (A.L.); agnieszka.herosimczyk@zut.edu.pl (A.H.); 3 Department of Animal Physiology and Biochemistry, Poznan University of Life Sciences, 60-637 Poznan, Poland; pawelbigi@o2.pl

**Keywords:** in ovo technology, poultry, meat, synbiotics, gene expression

## Abstract

**Simple Summary:**

Poultry production plays a major role in providing meat products to global markets. Hence, there is a continued interest of researchers in the possibilities of improving the production parameters of broiler chickens. A direct response from muscles and their metabolism to in ovo synbiotic stimulation on day 12 of egg incubation has already been widely documented. However, it is necessary to analyze the molecular mechanisms determining the phenotypic effects. The present research aimed to explain the molecular background of the quality of broiler chicken meat after injection of synbiotics based on *Lactobacillus* strains into the air chamber of the egg. Characterization of the meat quality is based on the signature of the metabolic gene expression closely related to muscles and basic physiological parameters.

**Abstract:**

To better understand the effects of synbiotics administered at early stages of embryonic development in poultry, it is necessary to analyze direct effects (meat quality) and the molecular background. The molecular interpretation of poultry meat properties after in ovo administration of synbiotics remains to be reported. The purpose of the present study was to analyze the molecular background of meat quality based on gene expression and basic physiological parameters. Eggs were injected with (S1) *Lactobacillus salivarius* with galacto-oligosaccharides or (S2) *Lactobacillus plantarum* with raffinose family oligosaccharides. The pectoral muscle was collected at two time points (day 7 and day 42) and subjected to RNA isolation. Gene expression analysis was performed by RT-qPCR for a panel of eight genes associated with metabolism. The concentration of glucose and hormones (insulin, glucagon, and leptin (S1 *p* = 0.04)) was also increased. The obtained results showed that metabolic gene expression in the muscle was more differential due to synbiotic stimulation on day 7 (*FST* in S1 *p* = 0.03; *PDK4* in S1 *p* = 0.02 and S2 *p* = 0.01; *CEBPB* in S1 *p* = 0.01 and S2 *p =* 0.008; PHKB in S1 *p =* 0.01; *PRKAG3* in S1 *p =* 0.02) than on day 42 (*PDK4* in S1 *p =* 0.04). On the basis of the results obtained, it can be concluded that in ovo stimulation with S1 triggered the most potent and favorable changes in the pectoral muscle gene expression in broiler chickens.

## 1. Introduction

Poultry production plays a key role in delivering high-quality meat to global markets. Consumption of poultry meat has increased in recent years; hence, the prices of poultry meat, especially chicken broilers, are much more competitive than those of other species [1]. Consumer demands have led to refining of the genetics, production, and feeding technology of chicken broilers [2]. Currently, commercial chicken broiler lines are genetically selected to achieve maximum productivity. The quality and composition of the meat are also affected by the treatment of birds during rearing and by supplementation with bioactive substances. It has already been proved that the administration of bioactive substances such as synbiotics [3], probiotics [4], and prebiotics [5] affects the rearing parameters and intestinal microbiota, which may indirectly affect meat quality. The gastrointestinal microbiota affects the host organism by regulating immune response, metabolism, digestion, and nutrient absorption [6]. A technology for reprogramming the composition of chicken microbiota at an early stage of embryonic development was developed by administering prebiotics or synbiotics using in ovo technology on day 12 of egg incubation [7,8]. The in ovo administered bioactive substances such as prebiotics like galacto-oligosaccharides (GOS) and inulin and synbiotics like *Lactococcus lactis* subsp. *cremoris* with GOS, *Lactococcus lactis* subsp. *lactis* with inulin, *Lactobacillus salivarius* with GOS, and *Lactobacillus plantarum* with raffinose family oligosaccharides (RFO) directly affect the composition of the microbiota, indirectly improve many physiological and production traits of chickens [8,9,10,11], and modulate gene expression in the spleen, cecal tonsils, large intestine, jejunum, and liver [8,12,13].

The effect of in ovo administration of synbiotics on gene expression in broiler muscle has not yet been reported. The direct effect of the administration of bioactive substances on muscle quality and structure in broiler chickens has been extensively described [9,14,15]. It was demonstrated that the administration of *Lactobacillus* synbiotics in ovo does not adversely affect the body weight of broiler chickens as well as the physicochemical properties of meat, cholesterol content, and intramuscular collagen properties [15]. These results may suggest that the administration of a bioactive substance does not elicit a strong defensive response in the organism. It is proved that a highly active immune system is very energy-consuming, which can negatively affect animal performance [16]. Synbiotics also reduce lipid content in breast meat, which can have a positive effect on human health [15]. A favorable fatty acid profile has also been indicated to have a beneficial effect on the nutritional properties of chicken broiler meat [15]. The synbiotics used in this experiment were constructed based on in vitro tests [8] and subjected to transcriptomic analysis in metabolic (liver), immune (spleen, cecal tonsils), and intestinal (jejunum) tissues [13]. The administration of synbiotic 1 (S1) composed of *Lactobacillus salivarius* with GOS showed strong activation of genes related to immune responses in cecal tonsils in microarray analysis. In chickens that received synbiotic 2 (S2) composed of *Lactobacillus plantarum* with RFO, genes associated with metabolic and developmental signaling pathways in the liver were modulated [13]. The administration of the *Lactobacillus* synbiotic on day 12 of egg incubation does not adversely affect the hatching and weight gain of chickens [8].

A direct response from muscles and their metabolism to synbiotic administration has been widely described; however, it is necessary to analyze the molecular mechanisms that determine such effects. This study aims to explain the molecular background of chicken meat quality after synbiotic injection into an air cell on day 12 of egg incubation, based on the expression signature of metabolic genes and basic physiological parameters.

## 2. Materials and Methods

### 2.1. In Ovo Injection of Lactobacillus Synbiotics

The method of synbiotic selection, dose optimization, and in ovo injection of *Lactobacillus* synbiotics has been described by Dunislawska et al. [8]. The experiment was conducted on 2400 roosters (Cobb500FF, Cobb Vantress B.V., Boxmeer, Holland) distributed randomly into three experimental groups: two groups were injected on day 12 of egg incubation with a synbiotic: S1—*Lactobacillus salivarius* with GOS and S2—*Lactobacillus plantarum* with RFO [17], and the control group was injected with 0.2 mL of physiological saline (0.9%). The experiment was conducted in a commercial hatchery (Drobex-Agro, Solec Kujawski, Poland) and a commercial farm (Piast, Olszowa Experimental Unit 0161, Poland).

### 2.2. Sample Collection and RNA Extraction

Randomly selected individuals from each experimental group and the control group were sacrificed in the first week of hatching (day 7) and at the end of the production cycle (day 42). Samples of pectoral muscle were collected from five individuals per group into 5 mL Eppendorf tubes containing an RNA stabilization buffer (fixRNA, EURx, Gdansk, Poland) and stored for further analysis. Tissues were homogenized with a homogenizer (TissueRuptor, Qiagen GmbH, Hilden, Germany) in 1 mL of Trizol (TRIzol LS Reagent, Ambion/Thermo Fisher Scientific, Valtham, USA). The obtained homogenates were additionally cleaned on-column with a commercial kit (Universal RNA Purification Kit, EURx, Gdansk, Poland). Quality and quantity of the isolated RNA were assessed by electrophoresis and spectrophotometry (NanoDrop 2000, Scientific Nanodrop Products, Wilmington, NC, USA). Blood was collected from eight individuals per group on day 7 and day 42, and blood serum was obtained for hormonal profile analysis. The materials (RNA and blood serum) were frozen and kept at −80 °C for further investigations. Animal use for experiments was approved by the Local Ethical Committee for Animal Experimentation, University of Science and Technology, Bydgoszcz, Poland (Approval No. 36/2012 on 12 July 2012).

### 2.3. Gene Selection and Primer Design

The gene panel was selected on the basis of the literature data. The genes were identified according to their biological functions related to metabolism: lipid metabolism, muscle growth regulation, and meat quality [18]. Sequences of primers were based on the literature [18]. Primer sequences are shown in Table 1.

### 2.4. RT-qPCR Reaction

After RNA isolation, cDNA was synthesized using the Maxima First Strand cDNA Synthesis Kit for RT-qPCR (Thermo Scientific/Fermentas, Vilnius, Lithuania) following the manufacturer’s recommendations. The obtained cDNA was diluted to 70 ng/μL. The reaction mixture included Maxima SYBR Green qPCR Master Mix (Thermo Scientific/Fermentas), 1 μM of each primer, and 140 ng of diluted cDNA. Thermal cycling was performed in a LightCycler II 480 (Roche Diagnostics, Basel, Switzerland). Each RT-qPCR reaction was conducted in two technical replicates. Gene expression analysis was performed for the selected panel of genes: *FST, RGS2, PDK4, CEBPB, PHKB, PRKAG3, ACSL1,* and *ABHD5*.

### 2.5. Relative Quantification of Gene Expression

Relative gene expression analysis was performed for each experimental group by the ddCt [22] method using *UB* and *G6PD* as reference genes. Geometric means of cycle threshold (Ct) values of reference genes were used in the analysis [23]. Ct differences between target and reference genes were calculated for each sample. Control samples (injected with saline) were used as calibrators. Statistical analyses were performed by comparing the Ct value of each experimental group with the control group by Student’s *t*-test (*p *< 0.05).

### 2.6. Hormonal and Biochemical Profiles

The blood serum hormone concentration was analyzed using commercial RIA and EIA kits: insulin (Insulin RIA kit, Millipore, Billerica, Massachusetts, USA), leptin (Multi-Species Leptin RIA, Millipore), glucagon (EIA kit, Phoenix Pharmaceuticals Inc., Burlingame, CA, USA) as described by Kolodziejski et al. [24]. To verify the binding specificity of the kits, a serum dilution curve was performed. For this purpose, serum from five randomly selected individuals per group was diluted 2 times, 4 times, and 10 times in ELISA/RIA buffer, and the concentrations of investigated hormones were determined in all dilutions. Glucose was measured using a Glucose Assay Kit (Pointe Scientific, Canton, Michigan, USA).

## 3. Results

### 3.1. Changes in the Metabolic Gene Expression in Muscles in Response to In Ovo Stimulation

**Figure 1:**
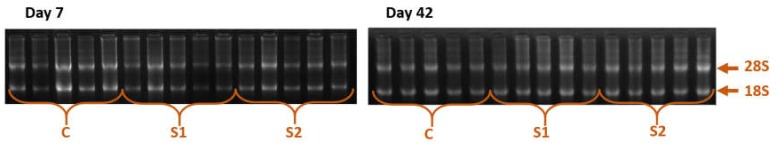
Assessment of RNA separation on 2% agarose gel. RNA was isolated from the pectoral muscle collected on days 7 and 42 from broiler chickens (3 groups per time point: C, S1, S2; *n* = 5).

The study was designed to analyze the effect of synbiotics injected in ovo on muscle transcriptome at the beginning and end of broiler life. Isolated RNA for expression analysis was measured using a Nanodrop Spectrophotometer. The concentration of RNA from samples was between 670 and 960 ng/µL. The A260/280 ratio was between 2.11 and 2.17, while the A260/230 ratio was between 2.08 and 2.19. The RNA integrity assessment performed using 2% agarose gel is presented in Figure 1. On this basis, RNA was qualified for further analysis.

**Figure 2:**
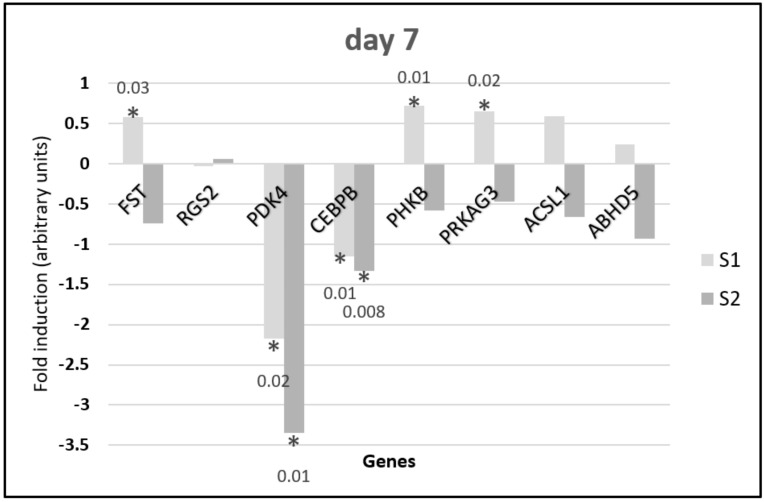
Changes in the relative expression of metabolic genes in the pectoral muscle of 7-day-old broiler chickens injected in ovo with synbiotics (S1) *Lactobacillus salivarius* with GOS or (S2) *Lactobacillus plantarum* with RFO. The analysis was performed using the ddCt method to determine gene expression changes (fold induction). Synbiotics were injected on day 12 of embryo development. Tissues were sampled on day 7 post hatching (*n* = 5). Statistical analysis consisted of comparing the experimental groups with the control group by Student’s *t*-test (* for *p* < 0.05; exact *p* value).

**Figure 3:**
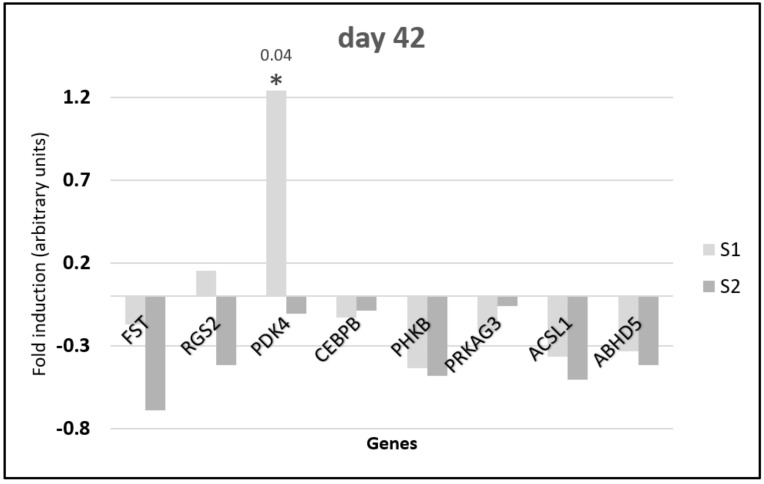
Changes in the relative expression of metabolic genes in the pectoral muscle of 42-day-old broiler chickens injected in ovo with synbiotics (S1) *Lactobacillus salivarius* with GOS or (S2) *Lactobacillus plantarum* with RFO. The analysis was performed using the ddCt method to determine gene expression changes (fold induction). Synbiotics were injected on day 12 of embryo development. Tissues were sampled on day 42 post hatching (*n* = 5). Statistical analysis consisted of comparing the experimental groups with the control group by Student’s *t*-test (* for *p* < 0.05; exact *p* value).

Quantitative PCR analysis showed changes in gene expression depending on the synbiotic delivered in ovo and the day of sample collection. Results for day 7 are shown in Figure 2, while those for day 42 are shown in Figure 3. Metabolic gene expression in the muscle was more differential due to synbiotic stimulation on day 7 than on day 42. On day 7 (Figure 2) the *FST, PHKB, PRKAG3, ACSL1*, and *ABHD5* genes showed the same direction of gene expression changes: upregulation after S1 stimulation and downregulation in response to S2 stimulation. The upregulation of *FST, PHKB*, and *PRKAG3* expression after S1 delivery was statistically significant (*p* < 0.05). Both synbiotics caused downregulation of *PDK4* and *CEBPR* expression. These changes were statistically significant. There were no significant changes in RGS2 expression.

On day 42 (Figure 3), all genes except *RGS2* and *PDK2* for S1 stimulation showed downregulation of expression. The upregulation of the *PDK4* gene expression after S1 administration was statistically significant.

On day 7, the expression of five genes was significantly altered following in ovo injection of S1 synbiotics when compared with the control group. Of these genes, an increased expression was observed for PRKAG3, PHKB, and FST, whereas the remaining two genes, namely *PDK4* and *CEBPB*, were downregulated. Interestingly, S2 synbiotic triggered changes in the expression of two genes (*PDK4* and *CEBPB*), and these genes followed the same direction of expression changes as observed in the group stimulated with S1.

### 3.2. Changes in Hormonal and Biochemical Profiles in Response to In Ovo Stimulation

**Figure 4:**
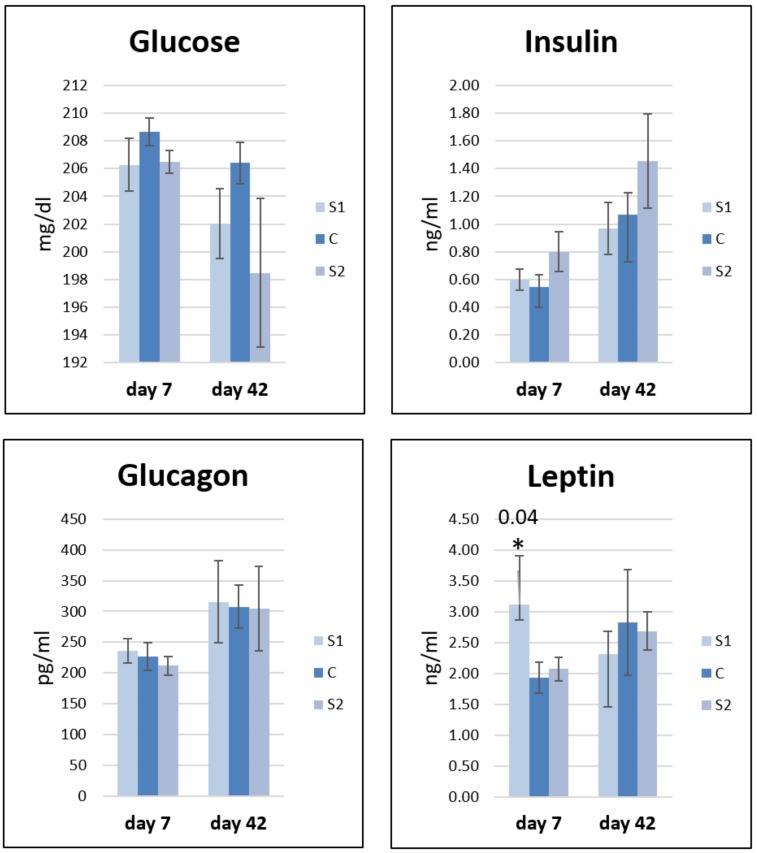
Changes in biochemical (glucose) and hormonal (insulin, glucagon, and leptin) profiles in response to an early stimulation in ovo with synbiotics (S1) *Lactobacillus salivarius* with GOS or (S2) *Lactobacillus plantarum* with RFO and saline as the control (C). Statistical analysis consisted of comparing the experimental groups with the control group by Student’s *t*-test (* for *p* < 0.05; exact *p* value). Standard errors of the mean (SEM) are marked with the lines; *n* = 8.

The concentration of glucose and hormones (insulin, glucagon, and leptin) was investigated in blood serum on day 7 and day 42 (Figure 4). The glucose level decreased in S1 and S2 as compared to that in the control group at both time points. On day 7, the insulin level increased in S1 and S2 relative to control, while on day 42, it decreased in S1 but continued to increase in S2. The glucagon level increased at both time points in S1 and decreased in S2. The leptin level increased on day 7 in S1 and S2, and the increase was particularly statistically significant after S1 administration. On day 42, the leptin level decreased in S1 and S2 relative to control.

## 4. Discussion

The present study aimed to explain the molecular background of chicken meat quality after *Lactobacillus* synbiotics administration in ovo based on gene expression changes and basic physiological parameters. As reviewed by Siwek et al. [25], in chickens hatched from eggs injected in ovo with synbiotics, some long-term beneficial effects were observed. These effects involved quantitative and/or qualitative alterations of the gut microbiota, increased formation of short chain fatty acids (SCFAs), and stimulation of the gastrointestinal tract (GIT) development, which in turn may change the pattern of gene expression in various tissues [25].

In our opinion, the trend of gene expression alterations might be explained by an increased production of SCFAs in the large intestine of broiler chickens after in ovo stimulation with both S1 and S2. Although cecal and colonic SCFA concentrations were not measured in the current study, it was previously demonstrated that in broiler chickens, both synbiotic [26] or probiotic administration [27] led to an increase in cecal acetic, butyric, and propionic acid content. Moreover, recent in vitro and in vivo studies showed that gut microbiota through its fermentation end products, especially acetate and butyrate, may activate the mechanisms involved in regulating energy metabolism in skeletal muscle [28,29]. These effects have been attributed to increased AMP-activated protein kinase (AMPK) phosphorylation, a protein known to be a major cellular energy sensor in the muscle tissue. The activation of skeletal muscle AMPK is mediated either directly by increasing the AMP/ATP ratio or indirectly through the free fatty acid receptor 2 (FFAR2)-leptin pathway [28,30,31]. The results of the current study support the abovementioned findings, as the upregulation of the *PRKAG3* gene, which encodes a muscle-specific isoform of the regulatory gamma subunit of AMPK, and significantly increased plasma leptin levels were observed in the group of birds stimulated in ovo with S1. Leptin, a hormone produced in both the liver and adipose tissue in chickens, has been shown to stimulate the oxidation of fatty acids through AMPK activation, and it also decreases the activity of mechanisms involved in intracellular lipid accumulation in muscles [32,33]. A previous study conducted on the same group of chickens showed that in ovo treatment with synbiotics caused lower total lipid content in breast muscles of 42-day-old chickens; however, this effect was significant in the case of S2 injection [15]. Moreover, SCFAs are also implicated in the regulation of blood glucose levels by increasing insulin secretion and decreasing the production of glucagon in response to SCFA-induced GLP-1 secretion [31,34]. The results of the current study revealed a trend toward lower plasma glucose and higher insulin levels on day 7 in birds from both experimental groups; however, these values did not reach statistical significance. Nevertheless, it seems that our data support the general idea that SCFAs are implicated in the activation of mechanisms leading to improved glucose homeostasis and insulin sensitivity. A substantial downregulation of the *PDK4* and *CEBPB* genes was observed in both synbiotic-treated groups. As shown by Connaughton et al. (2010), the transcription of the *PDK4* gene is inhibited by insulin, and its expression is maintained at a low level when the cell uses glucose instead of fatty acids as the primary energy source [35]. Furthermore, a decreased hepatic *CEBPB* expression was related to an increased insulin secretion from the pancreatic β-cells [36]. Our data also confirmed a reduced expression of the *PHKB* gene in both experimental groups. The *PHKB* gene encodes a phosphorylase kinase, a protein involved in glycogen breakdown through glycogen phosphorylase activation [37]. Recently, Dankowiakowska et al. (2019) revealed that in ovo injection of synbiotics (inulin and *Lactobacillus lactis*) caused a significant increase in the percentage of glycolytic fibers in the pectoral muscle in broiler chickens [14]. However, further research is needed to elucidate the underlying mechanisms of synbiotic action on the process of glycolysis in muscles. An upregulation of the *FST* gene found in the group of birds stimulated with S1 synbiotic may indicate an increase in muscle mass as this protein has been shown to stimulate muscle growth in mice [38]. It seems that a significantly increased plasma leptin level, shown in this group of birds, is responsible for the observed effects. A previous study reported that leptin administration in mice inhibits the expression of myostatin in skeletal muscle, a protein known to be a major inhibitor of myoblast proliferation and differentiation [39]. Therefore, we can assume that an increased expression of the *FST* gene, a potent antagonist of myostatin, may reflect an activation of the anabolic pathways leading to increase in muscle mass. However, this effect was not confirmed in birds at the 42 day of age as the pectoral muscle weight was not influenced in this group of chickens [15].

Surprisingly, on day 42 post hatching, increased *PDK4* expression was observed in the group of chickens in ovo injected with *Lactobacillus salivarius* with GOS. It seems very likely that this effect may be related to reduced plasma insulin levels when compared with the control groups. A previous study showed that a decreased insulin concentration suppresses glucose oxidation, which favors fatty acid uptake by the muscle cells through PDK4-mediated inhibition of the pyruvate dehydrogenase complex [40].

## 5. Conclusions

In conclusion, our results suggest that in ovo stimulation with *Lactobacillus salivarius* with GOS triggered the most potent and favorable changes in the pectoral muscle gene expression in broiler chickens. The genes found to be altered in the S1 group were mainly related to improved skeletal muscle energy metabolism, glucose homeostasis, and insulin sensitivity. Additionally, our data also revealed an increased plasma leptin levels, which triggered changes in the expression of genes involved in mechanisms leading to increased muscle mass accumulation with concomitant reduction in lipid accumulation in muscle tissue. However, it should also be noted that these changes were observed only in 7-day-old birds but not in the older ones. We do not have a solid explanation for this observation. However, we believe that our data will add to the existing knowledge on the mechanisms of action of synbiotics in chickens and may be valuable to the poultry industry.

## Figures and Tables

**Table 1:** Primer sequences used in the RT-qPCR reaction.

Symbol	Gene Name	Gene ID	Description	Primer Sequence (Forward (F)/Reverse (R))	Ref.
UB	Ubiquitin C	396425	reference gene; associated with DNA repair, protein degradation, cell cycle regulation, kinase modification, and regulation of cell signaling pathways	F: GGGATGCAGATATTCGTGAAAR: CTTGCCAGCAAAGATCAACCTT	[19]
G6PD	Hexose-6-phosphate dehydrogenase	428188	reference gene; catalyzes the rate-limiting step of the oxidative pentose-phosphate pathway, provides reducing power (NADPH) and pentose phosphates for fatty acid and nucleic acid synthesis	F: CGGGAACCAAATGCACTTCGTR: GGCTGCCGTAGAGGTATGGGA	[20]
FST	Follistatin	396119	binds directly to activin and functions as an activin antagonist; inhibits activin A signaling and regulates somatostatin phenotype; specific inhibitor of the biosynthesis and secretion of pituitary follicle-stimulating hormone (FSH)	F: AGGAGGACGTCAACGACAACR: TGGCAGATTCAGTTGCAAGA	[13]
RGS2	Regulator of G-protein signaling 2, 24 kDa	378912	regulates G protein-coupled receptor signaling cascades, inhibits signal transduction by increasing the GTPase activity of G protein alpha subunits, thereby driving them into their inactive GDP-bound form; involved in the negative regulation of the angiotensin-activated signaling pathway; plays a role in the regulation of blood pressure; binds EIF2B5 and blocks its activity	F: CTGACGCTGAAGGCAAAGAAAATCR: CAGAAACCGTGGGTACGAGTTG	[18]
PDK4	Pyruvate dehydrogenase kinase, isozyme 4	420570	kinase that plays a key role in the regulation of glucose and fatty acid metabolism and homeostasis via phosphorylation of the pyruvate dehydrogenase subunits; it inhibits pyruvate dehydrogenase activity and thereby regulates metabolite flux through the TCA cycle, downregulates aerobic respiration, and inhibits the formation of acetyl-coenzyme A from pyruvate; regulates both fatty acid oxidation and de novo fatty acid biosynthesis	F: TGACTGGTGCATCCCAAGTAAAGR: GGAAGAATTTGCCTGTTTGGAGG	[18]
CEBPB	CCAAT/enhancer binding protein (C/EBP), beta	396185	important transcriptional activator that regulates the expression of genes involved in immune and inflammatory responses; regulates the transcriptional induction of PPARγ; also plays a significant role in adipogenesis as well as in the gluconeogenic pathway, liver regeneration, and hematopoesis	F: GCAAGAACAAGCCCAAGAAGTGR: CAAGACTTTGTGCTGCGTCTCC	[18]
PHKB	Phosphorylase kinase regulatory subunit beta	415741	Phosphorylase b kinase catalyzes the phosphorylation of serine in certain substrates, including troponin I; the beta chain acts as a regulatory unit and modulates the activity of the holoenzyme in response to phosphorylation; involved in the glycogen metabolism pathway	F: GCTTAACCGACGACAAATAGATGGR: CGTCATATCCGATAAGGTTGGTTG	[21]
PRKAG3	Protein kinase AMP-activated noncatalytic subunit gamma 3	424208	AMP/ATP-binding subunit of AMP-activated protein kinase (AMPK), an energy sensor protein kinase that plays a key role in regulating cellular energy metabolism; in response to reduction of intracellular ATP levels, AMPK activates energy-producing pathways and inhibits energy-consuming processes: inhibits protein, carbohydrate, and lipid biosynthesis as well as cell growth and proliferation	F: CCGACAACAATTTCCAGAGCCR: TCTGCATCTTGCTGTCCCACAG	[21]
ACSL1	Acyl-CoA synthetase long-chain family member 1	422547	catalyzes the conversion of long-chain fatty acids to their active form acyl-CoAs for both synthesis of cellular lipids and degradation via beta-oxidation; preferentially activates arachidonate than epoxyeicosatrienoic acids (EETs) or hydroxyeicosatetraenoic acids (HETEs)	F: CCTTCGCTGCATTAACACAATTCCR: CCACATTCATCATGGGGAAAAC	[18]
ABHD5	Abhydrolase domain containing 5	420673	coenzyme A-dependent lysophosphatidic acid acyltransferase that catalyzes the transfer of an acyl group on a lysophosphatidic acid; functions in phosphatidic acid biosynthesis; may regulate the cellular storage of triacylglycerol through the activation of the phospholipase PNPLA2	F: TTTTACCAGGGCTGGGGAATGGR: AATGCACTAATCTGCTGTGGGTG	[18]
